# Evidence of superconducting Fermi arcs

**DOI:** 10.1038/s41586-023-06977-7

**Published:** 2024-02-07

**Authors:** Andrii Kuibarov, Oleksandr Suvorov, Riccardo Vocaturo, Alexander Fedorov, Rui Lou, Luise Merkwitz, Vladimir Voroshnin, Jorge I. Facio, Klaus Koepernik, Alexander Yaresko, Grigory Shipunov, Saicharan Aswartham, Jeroen van den Brink, Bernd Büchner, Sergey Borisenko

**Affiliations:** 1 Leibniz Institute for Solid State and Materials Research, IFW Dresden, Dresden, Germany; 2 Kyiv Academic University, Kyiv, Ukraine; 3 Helmholtz-Zentrum Berlin für Materialien und Energie, Berlin, Germany; 4 Centro Atómico Bariloche, Instituto de Nanociencia y Nanotecnología (CNEA-CONICET) and Instituto Balseiro, San Carlos de Bariloche, Argentina; 5 Max Planck Institute for Solid State Research, Stuttgart, Germany; 6 Würzburg-Dresden Cluster of Excellence ct.qmat, Dresden, Germany; 7 Present Address: Helmhotz-Zentrum Dresden-Rossendorf, Dresden, Germany

**Keywords:** Topological matter, Superconducting properties and materials

## Abstract

An essential ingredient for the production of Majorana fermions for use in quantum computing is topological superconductivity^1,2^. As bulk topological superconductors remain elusive, the most promising approaches exploit proximity-induced superconductivity^3^, making systems fragile and difficult to realize^4–7^. Due to their intrinsic topology^8^, Weyl semimetals are also potential candidates^1,2^, but have always been connected with bulk superconductivity, leaving the possibility of intrinsic superconductivity of their topological surface states, the Fermi arcs, practically without attention, even from the theory side. Here, by means of angle-resolved photoemission spectroscopy and ab initio calculations, we identify topological Fermi arcs on two opposing surfaces of the non-centrosymmetric Weyl material trigonal PtBi_2_ (ref. ^9^). We show these states become superconducting at temperatures around 10 K. Remarkably, the corresponding coherence peaks appear as the strongest and sharpest excitations ever detected by photoemission from solids. Our findings indicate that superconductivity in PtBi_2_ can occur exclusively at the surface, rendering it a possible platform to host Majorana modes in intrinsically topological superconductor–normal metal–superconductor Josephson junctions.

## Abstract (web summary)

We provide evidence for superconducting topological Fermi arcs in PbBi_2_—a Weyl semimetal previously studied mostly for its bulk properties—from which Marjorama fermions could be derived for research in quantum computers.

## Main

The realization of topological superconductivity in new materials, which leads to robust Majorana fermions, has so far been hindered by numerous experimental challenges. Among them are the sophisticated growth of nanowire single crystals and heterostructures, as well as fine-tuning of the composition of non-stoichiometric compounds. Additionally, the rarity of spin-triplet superconductors and extremely small inverted gaps in iron-based superconductors^7^, proposed as intrinsic heterostructures^10^, are responsible for the lack of success in existing materials.

Weyl semimetals bear non-degenerate spin states both in the bulk and at the surface and the doped version of either time-reversal-breaking or non-centrosymmetric Weyl semimetals can become superconducting^11^. The search for topological superconductivity in such systems has been focused on finding bulk superconductivity, which would lead to Majorana fermion surface states. The possibility of intrinsic superconductivity of the arcs themselves, related to the topology of the band structure with Weyl nodes, has virtually not been considered. Although the arcs cannot support superconductivity in time-reversal-breaking Weyl semimetals^1^, the non-centrosymmetric varieties remain an option. Indeed, very recently, superconductivity associated with only the Fermi arcs of such systems has been predicted theoretically^12^.

Trigonal PtBi_2_ has emerged recently as a type-I Weyl semimetal that reportedly exhibits superconductivity^9,13^, making it an attractive candidate for topological superconductivity. Scanning tunnelling spectroscopy experiments confirmed the presence of surface superconductivity by observing typical spectra of superconducting gaps^13,14^. These spectra provided the evidence that the topological Fermi arcs bear the superconductivity in PtBi_2_. This occurs on both of the non-equivalent surfaces of PtBi_2_.

## Three-dimensional band structure

**Fig. 1:**
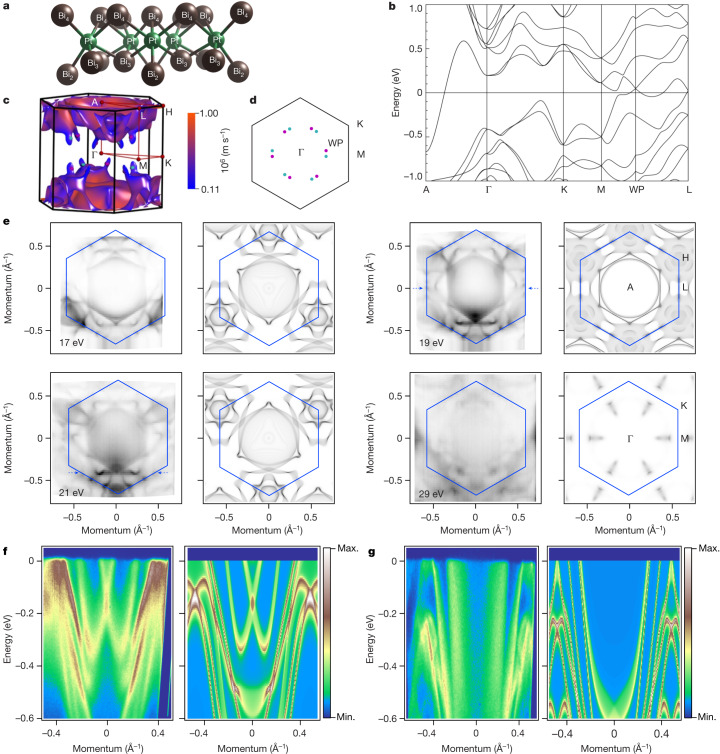
3D band structure of PtBi_2_. **a**, Crystal structure of PtBi_2_. **b**, Fragment of the band structure. One Weyl point is included. **c**, Fermi surface, Weyl and high-symmetry points. Colour scale indicates Fermi velocity. **d**, ΓMK plane of the Brillouin zone with projections of the Weyl points. Magenta (blue) colours stand for positive (negative) chirality. **e**, Fermi surface maps taken using different photon energies and the corresponding results of the band structure calculations. We note that fixed photon energy probes a sphere of the large radius in the *k*-space, matching theoretical data formally only at one point in the centre. Theoretical Fermi maps were averaged over a range of 1/10 of the Brillouin zone size in the *k*_z_ direction to account for experimental uncertainties. The intensities of the theoretical Fermi maps were normalized to the density of states for different *k*_*z*_ points. **f**,**g**, Left, energy-momentum intensity distributions at 21 eV (**f**) and 19 eV (**g**) along the cuts indicated by blue dashed arrows in **e**. Right, corresponding energy-momentum spectra taken from the band structure calculation. Source Data

**Extended Data Fig. 1:**
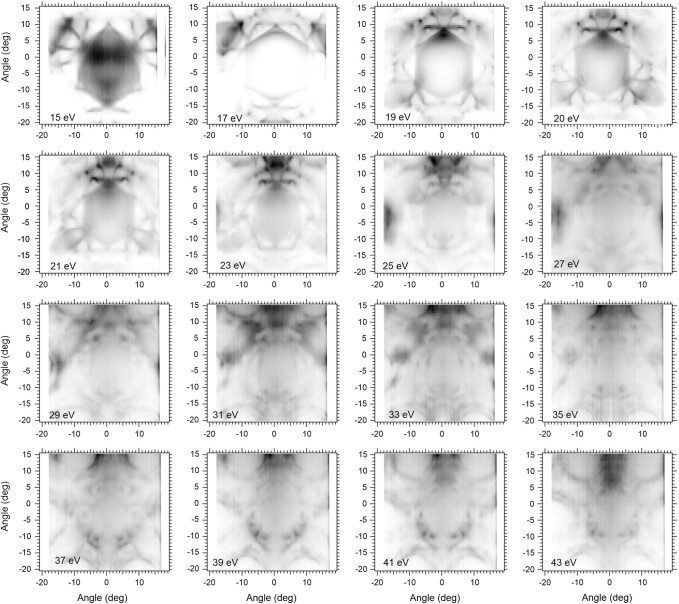
Fermi surface maps. Photoemission intensity integrated within a small energy region around the Fermi level. Data were recorded using 16 different photon energies.

The electronic structure of trigonal PtBi_2_ has been studied both experimentally and theoretically^9,15–19^. The material crystallizes in the trigonal *P*31*m* space group^13^ and exposes two different surfaces upon cleaving, which we refer to as A and B below (Fig. 1a). The band structure (Fig. 1b) arises mostly due to hybridization of Bi 6*p*, Pt 5*d* and Pt 6*s* states. Two sets of Weyl points are located in momentum space as shown in Fig. 1c,d, having the energy of 47 meV above the Fermi level. To set a baseline from which the three-dimensional (3D) band structure can be resolved, we have recorded 16 angle-resolved photoemission spectroscopy (ARPES) datasets covering at least the first 3D Brillouin zone and approximately 1 eV in energy using the photon energies from 15 to 43 eV (see also Extended Data Fig. 1). This allowed us to identify high-symmetry points along the *k*_z_ direction and find the value of the inner potential (*V*_0_ = 10.5 eV). In Fig. 1e we show the Fermi surface maps taken using the photon energies corresponding to high-symmetry points along the *k*_z_ direction and approximately half-way in between them. The latter two (left column) are easy to recognize by the pronounced *C*_3_ symmetry of the pattern, rotated by 60° with respect to each other. The map taken with 19 eV photons has a higher degree of hexagonal shape, which corresponds to the A-point of the Brillouin zone. The map taken with 29 eV photons at the level of the Γ-point bears a certain resemblance to the almost featureless calculated intensity as suggested by Fig. 1c. The experimental pattern in this case is connected with the finite *k*_z_ resolution of ARPES. In Fig. 1f,g, we also show the comparison of the dispersions along the lines indicated in Fig. 1e. The features look very similar, being shifted in energy or momentum without any signatures of strong renormalization, or similar manifestations (see also Extended Data Fig. 3). These data suggest a reasonable general agreement between experiment and theory, which is in accord with previous ARPES studies^15–18^. The experimental confirmation of the main features of the band structure and Weyl points near the Fermi level thus implies that PtBi_2_ is indeed a Weyl semimetal, which we will fully confirm below.

## Surface states on two terminations

**Fig. 2:**
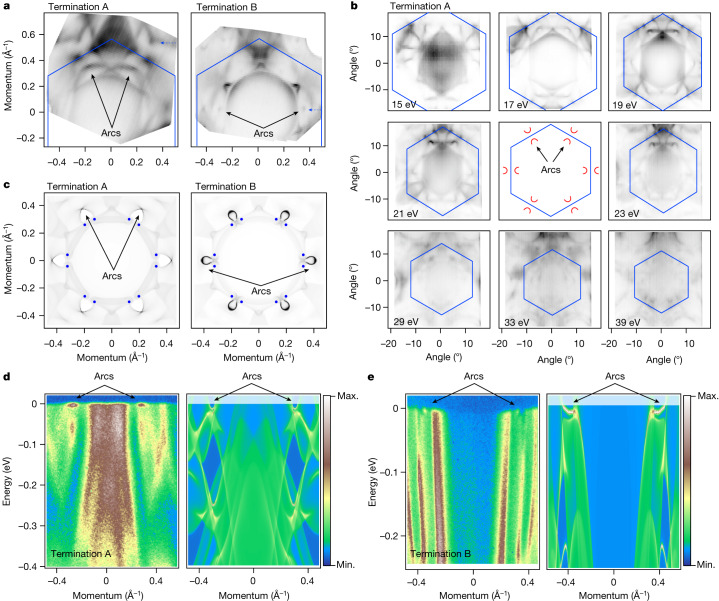
Fermi arcs. **a**, High-resolution Fermi surface maps (*hν* = 17 eV, *T* = 1.5 K) from both terminations. Arcs in the first Brillouin zone are indicated by the arrows. Note their presence in the equivalent positions in the first and repeated Brillouin zone. **b**, Fermi surface maps at different photon energies, all showing the presence of the arcs measured at 15 K. The sketch in the middle provides a visual reference for position of the arcs. **c**, Arcs as seen in the calculations. Blue dots show the projections of the Weyl points. **d**,**e**, Experimental and calculated energy-momentum intensity plots for terminations A (**d**) and B (**e**) along the cuts through the arcs highlighted by blue dashed arrows in **a**. Source Data

In Fig. 2a we present high-resolution Fermi surface maps from both terminations. Although seemingly different, closer inspection suggests that they share mostly the same pattern, provided intensity variations are taken into account. The number of localized features can be clearly distinguished in the map from termination B, at approximately 3/4 of ΓM distance and equivalent locations. These features have been overlooked in earlier ARPES studies^15–18^. Since the calculated bulk continuum displayed in Fig. 1b does not contain any similar electronic states in this region, we consider those as originating from the surface. The termination A map also shows similarly located features, but they are more clearly seen in the second Brillouin zone. The underlying bulk-related intensity is higher, masking the surface states. To establish their presence unambiguously, we show eight Fermi surface maps taken using different photon energies in Fig. 2b. All maps exhibit all the above features at the same location—approximately 3/4 of the ΓM distance, as in the case with termination A. Since it is unlikely that a particular bulk feature would be present in all of the recorded Fermi surface maps for a material with a highly 3D electronic structure, we conclude that these features also represent the surface. The schematic plot (in the middle of Fig. 2b) summarizes our observations regarding the locations of the arcs made from considering the Fermi surface maps.

Detected spots of intensity, which we identified above as surface states, remarkably coincide with the results of calculations which take into account the presence of the surface (Fig. 2c). Since PtBi_2_ is a Weyl semimetal, one does expect the presence of the topological Fermi arcs, different for terminations A and B. In an ideal type-I Weyl semimetal, the location of the starting and end points of the arcs should be identical as those are the projections of the Weyl points (Fig. 1d). The Weyl points, non-degenerate crossings of the bands in 3D *k*-space, are almost impossible to detect by ARPES directly because of the finite resolution, but the corresponding Fermi arcs have been repeatedly seen experimentally in various materials^20–24^.

Figure 2d,e demonstrates a comparison of the intensity distribution along the paths marked in Fig. 2a, which run through the arcs. The arcs are situated very close to the Fermi level and are well distinguished from the regions smeared out by *k*_*z*_-resolution bulk dispersions.

Considering the discrepancies in the experimental and theoretical 3D band structure (Fig. 1), we do not expect exact correspondence between the calculated Fermi arcs and the ARPES data, but the observed agreement proves not only that the experimental features are indeed the topological Fermi arcs, but also that PtBi_2_ is a Weyl semimetal.

## Robust Fermi arcs from laser-ARPES

**Fig. 3:**
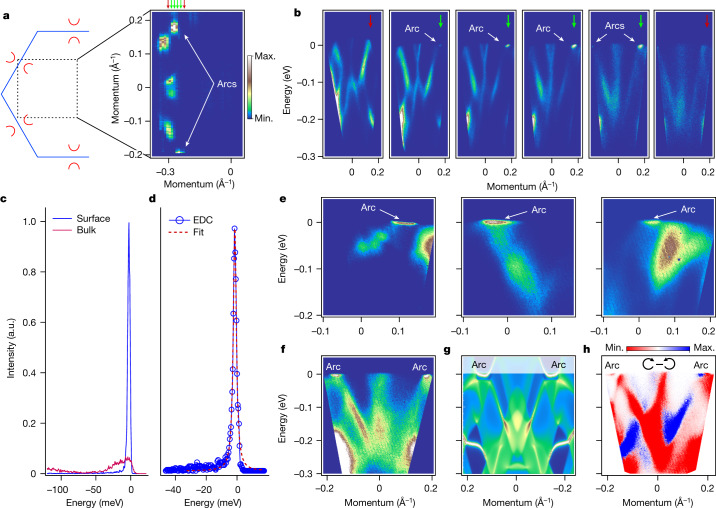
Laser-ARPES. **a**, Fermi surface map taken using *hν* = 5.9 eV at 3 K. Arcs are seen together with other bulk-originated features. **b**, Underlying dispersion along the momentum cuts indicated by arrows in **a**. **c**, Typical EDCs from **b**. Bulk EDC is taken close to zeroth momentum, while surface EDC corresponds to the arc. **d**, One of the narrowest and strongest EDCs detected in the present study. **e**, Arcs seen along the different cuts through the Brillouin zone in different experimental geometries. **f**, Intensity distribution taken using horizontally polarized light along the path crossing two arcs. **g**, The same momentum and energy range as in **f**, from the calculations. Note, the surface states at around 200 meV binding energies are also reproduced. **h**, Circular dichroism from the same region of the *k*-space. Colour bar in pannel **a** also applies to panels **b**,**e**,**f**, and **g**. Source Data

To study the detected spots of intensity in the Fermi surface maps in more detail, we carried out ARPES experiments using a laser setup. Because of the low kinetic energy of photoelectrons (approximately 1.7 eV), the part of the Brillouin zone that is accessible during these experiments is very limited. We have concentrated on detecting at least one arc in the portion of the *k*-space marked in the sketch of Fig. 3a. Several representative cuts through the key features seen in the map are shown in Fig. 3b. With that effort, the arc is better resolved, but still very localized in terms of both momentum and energy. We estimate the momentum extension to be of the order of 0.04 Å^−1^, which is in excellent agreement with theory (Fig. 2c).

The most striking characteristic of the arc states is their energy distribution. In Fig. 3c, we compare the energy distribution curves (EDCs) corresponding to the bulk and surface states. As the data are taken with very high resolution and at extremely low temperature, the Fermi momentum (*k*_F_) EDC representing bulk states has a well-defined maximum (full-width at half-maximum (FWHM), 30 meV) near the Fermi level and leading-edge width of approximately 5 meV. However, the sharpness and peak-to-background ratio of the EDC representing the Fermi arc is unprecedented. We have routinely observed the peaks having FWHM below 3 meV and a peak-to-background ratio of approximately 50 in numerous cleaves of many samples (Extended Data Fig. 4b and Methods). One such curve is shown in Fig. 3d. As far as we are aware, such a sharp peak has never before been observed in any photoemission experiment from solids.

In Fig. 3e, we show further appearances of the arcs in the momentum–energy plots from different cleaves and different terminations. The sharpness and flatness retain the robust characteristics of the feature in all our experiments at the lowest temperatures. We noticed that for A and B surfaces, the arc states are supported by the strongly and weakly dispersing bulk states, respectively, exactly as expected from theory.

Direct comparison with the calculations, considerating the presence of the topological surface states, is presented in Fig. 3f,g. The agreement with the experiment is remarkable: bulk- and surface-related dispersions are captured not only qualitatively but also quantitatively. The difference between the spectra taken with right- and left-circularly polarized light (Fig. 3h) allows us to identify additional features in the intensity distribution, making the agreement with the theory even stronger (see also Extended Data Fig. 6).

Despite the clear correspondence between laser-ARPES data and density functional theory calculations, there is one detail which remains unexplained—the striking flatness of the surface band without any signature of the Fermi level crossings.

## Superconductivity at the surface

**Fig. 4:**
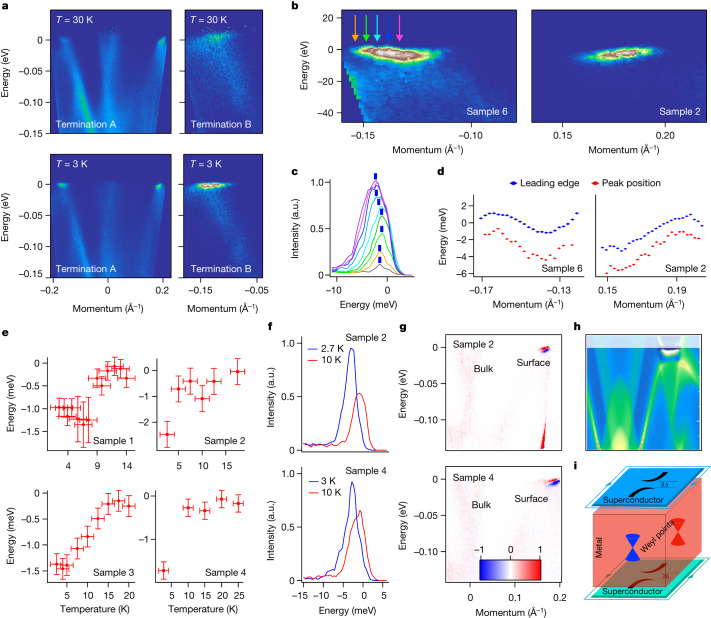
Superconducting arcs. **a**, Temperature dependence of the arcs’ dispersion from the terminations A and B. **b**, Zoomed-in datasets showing underlying dispersion of the arcs. **c**, EDCs corresponding to the coloured arrows in **b**. **d**, Leading edge and peak positions from **b**. **e**, Averaged values of the peak positions closest to the Fermi level as a function of temperature for different samples and terminations. Samples 1 and 3 correspond to termination A and Samples 2 and 4 correspond to termination B. **f**, Shift of the EDCs with temperature. **g**, Difference plots showing the changes of the intensity as a function of temperature. **h**, Results of the calculated spectral weight, taking into account the superconductivity at the surface. **i**, Schematics of the electronic structure of PtBi_2_. Green contours represent the Majorana states suggested by the topological superconductivity at the surfaces. a.u., arbitrary units. Source Data

**Extended Data Fig. 2:**
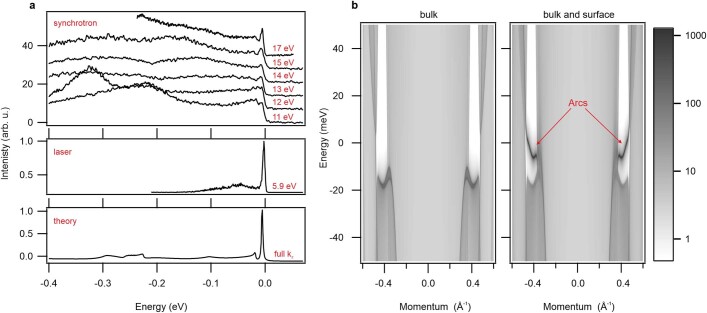
Photon energy dependence of the Fermi arcs. a, Energy distribution curves (EDC) across the Fermi arc as a function of photon energy (upper panel), EDC across the Fermi arc measured with 5.9 eV laser (middle panel), theoretical EDC for fully integrated bulk (lower panel). b, Theoretical calculation for bulk (left) and bulk with surface (right) in ΓM direction.

Record-high sharpness of the arc EDCs strongly resembles coherence peaks in ARPES data from superconductors (for example, ref. ^25^). To determine whether the electronic states in question bear any other characteristic features of superconductivity, we have carried out temperature dependent measurements. In Fig. 4a we show the datasets recorded at 3 and 30 K for both terminations. The comparison of the spectra taken at different temperatures underlines their flatness at the lowest temperature. The arcs clearly lose spectral weight and gain dispersion—just as is to be expected when the system enters the normal state. The apparent asymmetry of the arcs’ dispersion stems from the openness of the Fermi contour made by an arc—conventional electron-like Fermi surface pockets would be supported by the symmetric (with respect to the bottom) dispersions crossing the Fermi level. Extended Data Fig. 2b presents a zoomed-in picture of the calculated arcs, together with bulk bands. The arcs intersect the Fermi level on one side only and merge into the bulk zones on the other side, creating an open contour on the Fermi surface map.

We have also reproducibly observed another peculiar aspect of the superconducting state behaviour. In Fig. 4b, where the arcs are measured with the highest resolution, the typical back-bending of the dispersion from the side where the states most closely approach the Fermi level is clearly seen. This is illustrated in Fig. 4c,d where we plot EDCs at several momentum values as well as their peaks and their leading-edge positions. We further track the behaviour of the peak positions as a function of temperature in Fig. 4e. Actual EDCs corresponding to the broadest transition in sample 3 can be found in Extended Data Fig. 4b (Methods). Typical for superconductivity, shifts are observed when going through the critical temperatures *T*_c_. Such shifts measured at the *k*_F_ give a rather precise estimate of the superconducting gap *Δ*. Our measurements yield *T*_cA_ = 14 ± 2 K and *T*_cB_ = 8 ± 2 K, whereas the corresponding superconducting energy gaps are 1.4 ± 0.2 meV and 2 ± 0.2 meV, respectively. The transition for termination A seems to be broader and *T*_c_ higher compared to termination B, which suggests that slightly differing superconducting states set in on the opposing surfaces. Taking into account the different electronic structure of the two surfaces with Fermi arcs, it is not surprising that the superconducting orders are not fully equivalent. Signatures of Berezinskii–Kosterlitz–Thoules transition seen by transport^9^ may explain the unusual *Δ*/*T*_c_ ratios.

In Fig. 4f,g, we present additional evidence for essentially surface-related superconductivity observed in two different samples. While the EDCs corresponding to the surface states are clearly shifted with varying temperature (Fig. 4f), the bulk-related spectral weight remains virtually intact, showing only weak changes caused by the slightly different width of the Fermi function. This is illustrated with the aid of two-dimensional difference plots (Fig. 4g), where the clearly stronger variations of spectral function occur in the region where the arcs are located.

We can reproduce the experimental spectral function of PtBi_2_, including both the flatness and back-bending of the surface states, by switching on superconductivity only at the surface via a solution of the Bogoliubov–de Gennes (BdG) Hamiltonian for a semi-infinite solid with a gap function of *V*_0_ = 2 meV in the first three PtBi_2_ layers. The result is shown in Fig. 4h for the momentum and energy intervals corresponding to Fig. 4g (see also Extended Data Fig. 5 (Methods)). Note that only the electron–electron part of the BdG spectral density is plotted to model the ARPES signal and that only the states around the Fermi arcs acquire a gap at the Fermi level.

The superconductivity of arcs in PtBi_2_ follows not only from the emergence of unusually strong and sharp coherence peaks at low temperatures, flatness and back-bending of the dispersion, as well as characteristic shifts of the EDCs; it follows also from the striking agreement with recent scanning tunnelling microscopy (STM) data^14^—results of another surface-sensitive experiment on the crystals from the same batch. There (see figure 3 in ref. ^14^) the authors observed typical tunnelling conductance of superconductors characterized by the superconducting gaps varying in space. Remarkably, the average value of the gap closely corresponds to the gap values determined by ARPES. The rather unusual considerable zero-biased conductance observed by Schimmel et al.^14^ now has a very natural explanation in terms of a bulk contribution which remains ungapped. As seen from the ARPES data, the integrated contribution from the states associated with the bulk can easily reach a noticeable fraction of the signal from the surface, despite the dominant intensity of the arcs. The spot size of the laser beam in our study is of the order of 0.1 mm. This explains the agreement between the determined gap values with averaged STM data and does not exclude the existence of higher *T*_c_ regions, the detection of which by ARPES would require an application of micro- or nano-variations of the technique.

PtBi_2_ emerges as a stoichiometric Weyl semimetal with possible surface-only superconductivity (Fig. 4i) and thus opens up a plethora of possibilities to manipulate topological and superconducting phases in a single material. For instance, by varying the thickness of the single crystal, one can obtain a tunable Josephson junction that is intrinsically topological due to the Weyl semimetal forming the weak link. Topological superconductivity at the surface also may generate Majorana states at the edges. In this context it is interesting to note that we observe that the momentum-independent spectral weight at the Fermi level (apparently seen, for example, in Fig. 2d), appears to be enhanced when probing regions of the surface with a number of terraces. Further studies are needed to unambiguously identify and control both the higher-*T*_c_ superconductivity and possible Majorana states in surfaces and edges of PtBi_2_ single crystals and nano-structures.

## Methods

### ARPES measurements

ARPES measurements were carried out on the 1^2^ and 1^3^ARPES endstations^26^ at BESSY II synchrotron (Helmholtz-Zentrum Berlin), as well as in the Leibniz-Institut für Festkörper und Werkstoffforschung Dresden (IFW) laboratory using the 5.9 eV laser light source. Samples were cleaved in situ at a pressure lower than 1 × 10^−10 ^mbar and measured at the temperatures of 15 K and 1.5 K at BESSY II and 3-30 K in the IFW laboratory. The experimental data were obtained using the synchrotron light in the photon energy range from 15 to 50 eV with horizontal polarization and laser light with horizontal and circular polarizations. Angular resolution was set to 0.2–0.5° and energy resolution to 2–20 meV. The findings from the experiments were consistent and reproducible across multiple samples.

The simultaneous presence of bulk non-superconducting and surface superconducting states hinders the detection of true coherence peaks with ARPES. Our experiments at the synchrotron, with energy resolution of the order of 5 meV, turned out to be insufficient to detect even the shifts of the leading edges of the corresponding arc peaks having FWHM of the order of 10 meV and peak-to-background ratio of approximately 5. This is because the arc states are always on top of the bulk continuum. Only by measuring with energy resolution of the order of 1–2 meV did we manage to observe sufficiently sharp peaks (Fig. 3c,d and Extended Data Fig. 4) and their sensitivity to temperature. The sharpest features need to be found on the surface.

A superconducting gap on the arcs is most likely anisotropic. We included error bars in Fig. 4e to show the influence of a small shift of the beam spot and thus slightly different emission angle. Taking into account the very high localization in momentum space, this could lead to probing a different part of the arc and thus different *k*_F_, where the superconducting gap is slightly different.

### Bulk band structure and Fermi arc position

In Extended Data Fig. 1, we show ARPES Fermi surface maps obtained using the photon energies from 15 eV to 43 eV. Relatively strong variation of the pattern suggests a reasonable *k*_*z*_-sensitivity of our experiment. We found the optimal value of the inner potential to be equal to 10.5 eV. This agrees with the previous study of Jiang et al.^17^.

In Extended Data Fig. 2, we present further evidence that our assignment of the surface and bulk features is correct. Extended Data Fig. 2a shows EDCs taken across the Fermi arc for different photon energies (from synchrotron and laser sources), alongside the theoretical EDC for the fully integrated *k*_*z*_. The peak corresponding to the Fermi arc remains clearly visible without any noticeable dispersion for different values of *k*_*z*_, whereas the peaks located further below the Fermi level disperse. Such absence of the dispersion is peculiar to the surface states.

**Extended Data Fig. 3:**
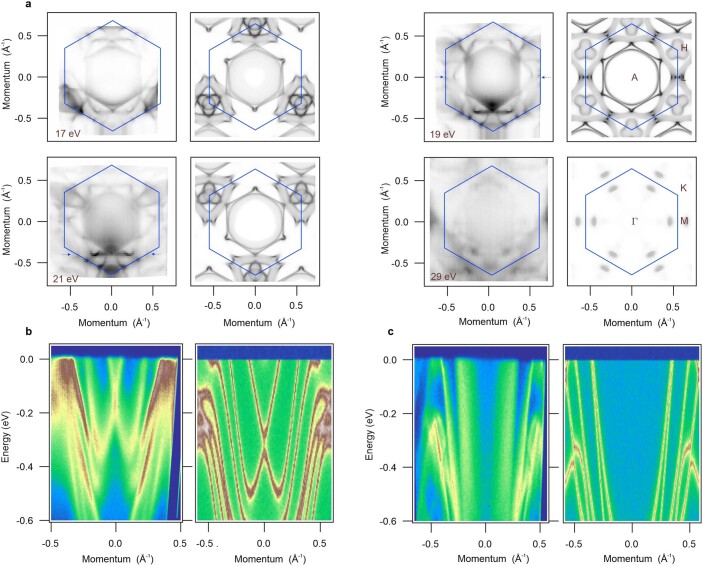
3D band structure. **a**, Fermi surface maps taken using different photon energies and corresponding results of the band structure calculations. We note, that fixed photon energy probes a sphere of the large radius in the *k*-space, matching theoretical data formally only at one point in the centre. **b**,**c,** Energy-momentum intensity distributions at 21 eV and 19 eV respectively along the cuts indicated by blue dashed arrows in panel **e**.

In Extended Data Fig. 3, we show an analogue of Fig. 1e–g, but here we compare experimental data with the results of band structure calculations carried out using the linear muffin-tin orbital (LMTO) method in the atomic sphere approximation as implemented in PY LMTO computer code^27^. As is seen from the figure, the agreement is at the same level as earlier, underpinning the previous conclusion as regards the good agreement between experimental and theoretical 3D band structure.

**Extended Data Fig. 4:**
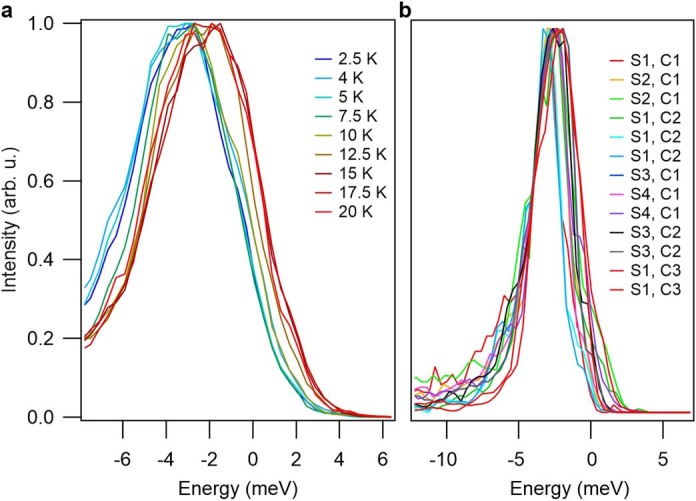
EDC across the Fermi arc. a, Energy distribution curves corresponding to sample # 3 from Fig. 4e of the main manuscript and normalized to the maximum intensity. The opening of the superconducting gap is clearly visible as a displacement of the peaks and leading edges with temperature. b, Energy distribution curves taken close to *k*_*F*_ of the arcs for different *k*, samples (S) and cleaves (C).

In Extended Data Fig. 4b, we present the sharpest EDCs from among the various samples and cleaves. Most have FWHM below 3 meV and a peak-to-background ratio of over 30.

### Band structure calculations

We performed density functional theory calculations using the full-potential nonorthogonal local-orbital scheme of ref. ^28^ within the general gradient approximation^29^ and extracted a Wannier function model. This allows determination of bulk projected spectral densities (without surface states) and the spectral densities of semi-infinite slabs via Green’s function techniques^30^. To model surface superconductivity of the semi-infinite slab, the Wannier model is extended into the BdG formalism with a zero-gap function except for a constant Wannier orbital diagonal singlet gap function matrix at the first three PtBi_2_ layers. A modification of the Green’s function method is used to accommodate this surface-specific term.

### Surface superconductivity calculations

To model a system which has a non-zero gap function only at the surface—in the first 30a_*B*_ which is 3(PtBi_2_) layers—we modified the standard Green’s function technique for semi-infinite slabs. The system is built by a semi-infinite chain of identical blocks consisting of 3(PtBi_2_) layers, repeating indefinitely away from the surface. Each block has a Hamiltonian *H*_*k*_ for each pseudo momentum *k* in the plane perpendicular to the surface and a hopping matrix *V*_*k*_, which couples neighbouring blocks. The blocks’ minimum size is determined by the condition that *H* and *V* describe all possible hoppings. To add superconductivity, the BdG formalism is used by extending the matrices in the following way:

$$\begin{array}{rcl}{H}_{k,{\rm{BdG}}} & = & \left(\begin{array}{cc}{H}_{k} & {\varDelta }_{k}\\ {\varDelta }_{k}^{+} & -{H}_{-k}^{* }\end{array}\right),\\ {V}_{k,{\rm{BdG}}} & = & \left(\begin{array}{cc}{V}_{k} & 0\\ 0 & -{V}_{-k}^{* }\end{array}\right),\end{array}$$

where we choose $${\varDelta }_{k}={\delta }_{i{i}^{{\prime} }}\left(\begin{array}{cc}0 & {V}_{0}\\ -{V}_{0} & 0\end{array}\right)$$ with *i* being a spinless Wannier function index and the 2 × 2 matrix to act in a single Wannier function’s spin subspace. This choice also leads to $\varDelta \left[{V}_{k,{\rm{BdG}}}\right]=0$, since *V* is an off-diagonal part of the full Hamiltonian. To model surface-only superconductivity, we let *V*_0_ = 0 for all (infinite) blocks, except the first one, which gets a finite *V*_0_ = 2 meV.

The standard Green’s function solution for this problem consists of determining the propagator *X* which encompasses all diagrams that describe paths that start at a certain block, propagate anywhere towards the infinite side of that block and return to that block. *X* also describes the Green’s function *G*_00_ of the first block and the self-energy to be added to the Hamiltonian to obtain *G*_00_ (a self-consistency condition) ${G}_{00}=X={\left({\omega }^{+}-H-\Sigma \right)}^{-1}$, Σ = *VXV*^+^ (in practice, however, self-consistency is obtained by an accelerated algorithm). From this recursion, relations can calculate all other Green’s-function blocks. These can be derived by subdividing propagation diagrams into irreducible parts using known components, in particular *X*.

If the first block differs from all the others (as is the case due to *Δ*_*k*_) one needs to modify the method in the following way. Let the first block have Hamiltonian *h* and hoppings to the second block *v* (while all other blocks are described by *H* and *V*). Then the irreducible subdivision of the propagation diagrams for *G*_00_ results in $g={\left({\omega }^{+}-h\right)}^{-1}$.

$$\begin{array}{l}{G}_{00}=g+gvX{v}^{+}g+(\,gvX{v}^{+})g\\ \,=\,\frac{1}{{\omega }^{+}-h-vX{v}^{+}}\end{array}$$

which contains the surface Hamiltonian and a modified self-energy depending on the *X* of the unmodified semi-infinite slab. From this we can derive the second block’s Green’s function

$${G}_{11}=X+X{v}^{+}{G}_{00}vX$$

and all others

$${G}_{n+1,n+1}=X+X{V}^{+}{G}_{nn}VX,\quad n > 0$$

which can be used to obtain the spectral density up to a certain penetration depth. Note that in our BdG case $H={H}_{k,{\rm{BdG}}}\left[{V}_{0}=0\right]$, $V={V}_{k,{\rm{BdG}}}\left[{V}_{0}=0\right]$ and $h={H}_{k,{\rm{BdG}}}\left[{V}_{0}\ne 0\right]$, *v* = *V*. The BdG spectral density is particle–hole symmetric and to obtain results that resemble ARPES data, one needs to use the particle–particle block *G*^ee^ (the upper left quarter of the *G* matrix) only.

**Extended Data Fig. 5:**
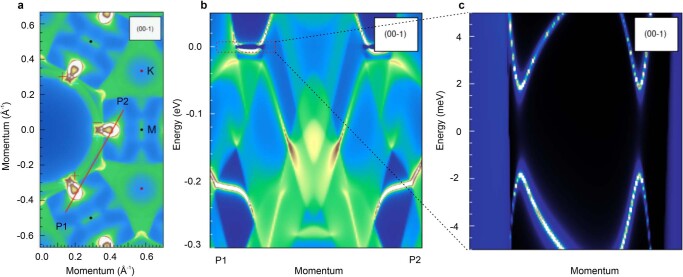
Theoretical calculations of gap opening in PtBi_2_. **a** The path in the BZ. **b** The surface-only superconducting spectral function of the (00-1)-surface for Δ = 2meV and penetration depth 30a_*B*_ (*G*_*ee*_ only). Note that the gap is only open around the surface state pocket. **c** the blow-up of this pocket. Note, that the gap is open for the surface state but closed for the bulk bands.

**Extended Data Fig. 6:**
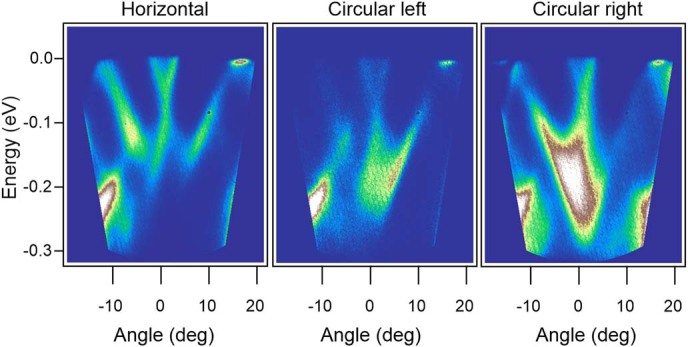
Polarization dependent datasets. ARPES spectra measured with horizontal (left), circular left (middle) and circular right (right) polarization of the 5.9 eV laser at 3.5 K.

Extended Data Fig. 5b shows the resulting spectra of this method along the path denoted in Extended Data Fig. 5a. Note that a gap is opened at the surface band pockets close to the Fermi energy, while the rest of the spectrum stays gapless (if we let *V*_0_ ≠ 0 for all blocks, we get a completely gapped spectrum). Extended Data Fig. 5c shows a zoomed-in region around the surface state. Note that the bulk bands are gapless (dark blue vertical features) while the surface state shows a gap and corresponding band back-bending. The particle–hole symmetry becomes apparent, although with a larger spectral weight for the occupied part because we use *G*^ee^ only.

### Further discussion

One approach to demonstrate the existence of topologically protected states with a topological insulator is to perform spin-resolved ARPES. In this technique, the spin-locking effect determines the spin structure in the vicinity of the surface Dirac node. However, the situation is quite different for Weyl semimetals. Here, there is no specific spin structure or configuration associated with the Weyl nodes, which can occur at generic points in the Brillouin zone. As inversion is broken and spin-orbital coupling present, each band at a generic *k*-point naturally possesses a spin direction, but this spin texture is smooth. Consequently, spin-resolved ARPES measurements cannot directly reveal Weyl points.

We would like to exclude the interpretation of our data based on density-wave order, which could, in principle, result in the similar features in the spectra. Charge density-waves require a redistribution of the spectral weight in the momentum space, characterized by the particular *k*-vector (vectors). We have always observed almost the same Fermi surface maps and underlying dispersions, independent of temperature. In line with these observations are the results of the STM studies which never detected any kind of a reconstruction. We have never observed any replica of the arcs or of the deeper lying surface states, such as a strong feature at (−0.2, −0.2) in Fig. 3f,g. It is also not clear which *k*-vector would be suitable for characterizing the density-wave order. If the arcs are simply superimposed in momentum, they all are of electron-like topology, so the opening of the hybridization gaps seems very unlikely. Finally, the fundamental difference between the density-wave gaps and superconducting gaps is that the latter are always pinned to the Fermi level. This is the only energy interval where we observe the changes in the spectra of PtBi_2_ with temperature.

## Online content

Any methods, additional references, Nature Portfolio reporting summaries, source data, extended data, supplementary information, acknowledgements, peer review information; details of author contributions and competing interests; and statements of data and code availability are available at 10.1038/s41586-023-06977-7.

### Source data

Source Data Fig. 1

Source Data Fig. 2

Source Data Fig. 3

Source Data Fig. 4

## Data Availability

Source data are provided with this paper. Other data are available from the corresponding authors upon reasonable request.
